# 肺黏液腺癌合并鳞状细胞乳头状瘤及惠普尔养障体感染1例

**DOI:** 10.3779/j.issn.1009-3419.2023.106.13

**Published:** 2023-07-20

**Authors:** Xiaolei YUE, Xiaoxia XI, Min ZHANG, Junyu BIAN, Yonglin CHEN

**Affiliations:** ^1^730000 兰州，兰州大学第一临床医学院; ^1^The First Clinical Medical School of Lanzhou University; ^2^兰州大学第一医院病理科; ^2^Department of Pathology, The First Hospital of Lanzhou University, Lanzhou 730000, China

**Keywords:** 肺肿瘤, 肺黏液腺癌, 鳞状细胞乳头状瘤, 惠普尔养障体, Lung neoplasms, Pulmonary mucinous adenocarcinoma, Squamous cell papilloma, Tropheryma whipplei

## Abstract

肺部同侧同时双原发性肿瘤且合并惠普尔养障体（Tropheryma whipplei, TW）感染较为少见，本文对1例原发性肺黏液腺癌（primary pulmonary mucinous adenocarcinoma, PPMA）合并支气管鳞状细胞乳头状瘤（bronchial squamous cell papilloma, BSCP）及TW感染患者的临床资料、影像学表现、病理结果及诊疗经过进行回顾，并分析诊疗经验。患者主要表现为长期咳嗽咳痰，胸部计算机断层扫描（computed tomography, CT）示双肺炎症改变与多发结节影，右肺下叶穿刺活检结果为PPMA；常规气管镜见右肺亚支结节，活检结果为BSCP。肺泡灌洗液高通量测序（metagenomics next generation sequencing, mNGS）结果考虑肺炎链球菌、TW混合感染，抗感染效果不佳。胸水未检测到明确基因突变，行化疗并定期随访。在临床中应该提高对肺部多发病变的认识，避免漏诊、误诊，尽快明确诊断后进行综合治疗。

## Abstract (en)

Simultaneous multiple primary tumors on the same side of the lung with Tropheryma whipplei (TW) infection are rare. We reviewed the clinical data, imaging manifestations, pathological results, diagnosis and treatment of a primary pulmonary mucinous adenocarcinoma (PPMA) patient with bronchial squamous cell papilloma (BSCP) and TW infection, and discussed our treatment experience. The patient mainly presented with chronic cough and sputum, and computed tomography (CT) showed inflammatory changes with multiple nodular shadows. Biopsy of the lower lobe of the right lung showed PPMA, and right lung sub-branchial nodules discovered during bronchoscope revealed BSCP. Metagenomics next generation sequencing (mNGS) of bronchoalveolar lavage fluid showed mixed infection of Streptococcus pneumoniae and TW with a poor anti-infective effect. No clear genetic mutation was detected, and the patient was treated with chemotherapy and regularly followed up. We should improve the awareness of multiple pulmonary pathologies during clinical practice, avoid missed diagnosis and misdiagnosis, and carry out comprehensive treatment after clarifying the diagnosis as soon as possible.

肺癌是发病率及死亡率最高的恶性肿瘤^[1]^，原发性肺黏液腺癌（primary pulmonary mucinous adenocarcinoma, PPMA）是肺腺癌中一种产生细胞内或细胞外黏液的少见亚型，约占肺腺癌的0.25%^[2]^。支气管鳞状细胞乳头状瘤（bronchial squamous cell papilloma, BSCP）是一种起源于鳞状上皮细胞的良性肿瘤，约占所有肺部肿瘤的0.38%^[3]^，其发生与高危型人乳头瘤病毒（high-risk human papillomavirus, HR-HPV）的感染密切相关。肺部同侧同时双原发性肿瘤较为少见，其病因及发病机制尚不明确，易与转移性肿瘤相混淆。本文报道1例肺同侧PPMA合并BSCP的罕见病例，并在诊治过程中意外发现惠普尔养障体（Tropheryma whipplei, TW）感染，旨在探讨其临床病理特征及经验，为疾病的早期诊疗提供依据。

## 1 病例资料

患者，男，68岁，主因“间断咳嗽、咳痰10年余，加重半月”就诊。患者10余年前无明显诱因咳嗽、咳白色泡沫状痰，未予重视或系统诊治，入院前半月余无明显诱因咳嗽、咳铁锈色痰，伴活动后胸闷、气短。既往糖尿病史10年余，阿尔兹海默病8年余，吸烟50年余，2包/天。体格检查：右下肺叩诊浊音，双肺可闻及细湿啰音。入院胸部计算机断层扫描（computed tomography, CT）（2022年2月14日）示双肺炎症改变并多发结节，右肺占位，右侧胸腔积液（图1A，图1B）。肿瘤标志物：细胞角蛋白19片段5.9 ng/mL（增高），癌胚抗原（carcinoembryonic antigen, CEA）4.6 ng/mL，鳞癌抗原1.0 ng/mL，神经元特异性烯醇化酶13.9 ng/mL。反复送检痰细菌涂片、细菌培养、抗酸染色、G试验、GM试验均阴性。超声示右侧胸腔50 mm的液性暗区，胸腔穿刺置管引流洗肉水样胸水230 mL，胸水常规及生化：浑浊，红细胞90×10^9^/L，白细胞760×10^6^/L，单个核细胞百分比94%，黏蛋白定性阴性，总蛋白58.7 g/L，碱性磷酸酶84.2 U/L，乳酸脱氢酶266 U/L，腺苷脱氢酶7.1 U/L，CEA 15.7 ng/mL，考虑为渗出液，恶性胸腔积液不排除，抗酸染色阴性。

**图1:**
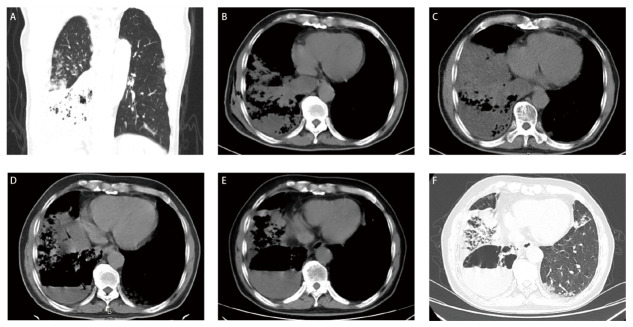
患者影像学检查的变化情况。A、B：患者入院时胸部CT（2022年2月14日）显示双肺散在斑片状磨玻璃影，右肺占位，右侧胸腔积液；C：抗感染治疗15天后复查CT（2022年3月1日）显示双肺感染并肺脓肿形成，右肺斑片影范围增大；D：随访复查CT（2022年6月14日）显示右肺空洞、气液平，渗出较前稍缩小；E、F：随访复查CT（2022年8月24日）显示双肺磨玻璃团片及结节灶，支气管黏液栓形成，右肺空洞。

为进一步明确病情，行常规气管镜检查，发现右肺上叶前支一亚支可见菜花样肿物（图2A），右肺下叶外后基底支分嵴处可见一小结节（图2B）。活检灰白色直径0.2 cm碎组织一堆，镜下观察：组织表面被覆鳞状上皮，细胞轻度不典型、乳头状增生，未见核分裂象或坏死，乳头中轴为纤维脉管束；免疫组化染色：细胞角蛋白5/6（cytokeratin 5/6, CK5/6）表达阳性，甲状腺转录因子-1（thyriod transcription factor-1, TTF-1）、天冬氨酸蛋白酶A（Napsin A）、神经细胞黏附分子（neural cell adhesion molecule, NCAM/CD56）、突触素（synaptophysin, Syn）和P16表达均阴性，P53表达阳性率约30%，增殖细胞核抗原（Ki-67）阳性率约5%；病理诊断：（右肺）鳞状细胞乳头状瘤（图2C）。送检肺泡灌洗液抗酸染色阴性，脱落细胞学检查未见异型细胞，高通量测序（metagenomics next generation sequencing, mNGS）检出肺炎链球菌序列数35,877，TW序列数26,172，人类疱疹病毒7型序列数142，蟾蜍分枝杆菌序列数83，粘质沙雷氏菌序列数68，结合临床考虑肺炎链球菌与TW混合感染。

**图2:**
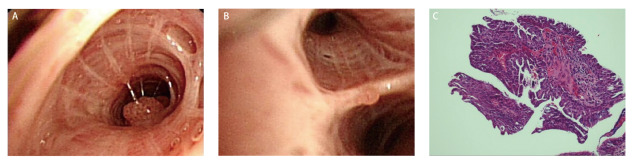
气管镜及活检结果。A、B：气管镜发现右肺上叶前支、右肺下叶外后基底支肿物；C：活检标本组织病理学检查显示，表面覆盖鳞状上皮，乳头中轴为纤维状血管束（HE染色，×40）。

患者入院以来经验性给予广谱抗生素厄他培南1.0 g/d，8天后（2022年2月22日）症状及肺部体征仍未明显改善，考虑抗感染效果不佳，遂在原抗炎的基础上加用哌拉西林钠他唑巴坦钠4.5 g/8 h。

结合胸水及影像学考虑恶性肿瘤可能性大，患者经评估后行CT引导下经皮右肺穿刺活检术（图3A），肉眼为灰白色0.5 cm-0.8 cm、直径0.1 cm组织三条，镜下观察：肺泡壁结构破坏，肺泡上皮被异型的黏液上皮取代，肿瘤细胞呈柱状，细胞质丰富，富含黏液，呈腺样、乳头状排列；免疫组化染色：TTF-1、细胞角蛋白7（cytokeratin 7, CK7）、绒毛蛋白（Villin）均阳性表达，CEA、Napsin A和细胞角蛋白20（cytokeratin 20, CK20）表达均阴性，P53表达阳性率约20%；病理诊断：（右肺）黏液腺癌（图3B，图3C）。入院15天后复查胸部CT（2022年3月1日）显示双肺感染并肺脓肿形成，右肺斑片影、结节较前增多，范围增大（图1C）。影像学提示患者抗感染效果仍不佳，患者最终诊断为：（1）右肺PPMA；（2）右肺BSCP；（3）恶性胸腔积液；（4）右肺肺脓肿并双肺肺炎；（5）2型糖尿病；（6）阿尔兹海默病。患者入院以来予抗感染、化痰、降糖、营养支持等综合治疗后一般情况尚可，咳嗽咳痰症状较前稍改善，胸水基因检测未检测到靶向用药相关基因突变，遂予“培美曲塞＋卡铂＋贝伐珠单抗”方案化疗。2个周期后患者因基础疾病不能配合治疗，2022年6月14日随访复查CT（图1D）显示右肺空洞、气液平，渗出较前稍缩小。试用口服安罗替尼靶向治疗（12 mg, qd），服2周休1周，每3周为1个周期。2022年8月24日随访复查CT（图1E，图1F）显示双肺磨玻璃团片及结节灶，支气管黏液栓形成，右肺空洞，考虑疾病进展。门诊末次随访时间为2023年3月5日，患者咳嗽、咳痰较前变化不大，失眠、淡漠症状加重，生活质量不佳。本文已获得患者及家属的知情同意。

**图3:**
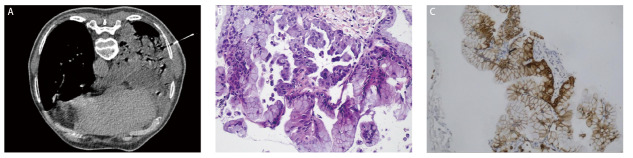
经皮右肺穿刺活检。A：CT引导下经皮肺穿刺活检；B：肿瘤细胞呈柱状，细胞质富含黏液，呈腺样、乳头状排列（HE染色，×100）；C：CK7免疫组化染色呈阳性（EnVision法，×100）。

## 2 讨论

### 2.1 概述

PPMA是起源于支气管上皮或黏膜下腺体具有多向分化潜能的干细胞，不同分化微环境下可分泌不同性质的黏液。根据国际肺腺癌多学科分类，PPMA包括黏液性原位腺癌、黏液性微浸润腺癌、浸润性黏液腺癌，其中浸润性PPMA符合下面一项或多项标准：大小（>3 cm）、浸润深度（>0.5 cm）、多发结节、粟粒状扩散到邻近肺实质而边界不清^[4]^。随着如低剂量螺旋CT、常规气管镜等诊断技术的广泛应用，早期肺癌、小结节型肺癌检出率不断提高，多原发肺肿瘤发病有增多趋势^[5]^，准确区分具有不同组织学类型的肺部结节仍是临床医生和病理医生面临的重要挑战。

TW是一种革兰阳性杆菌，其感染引起的惠普尔养障体病（Whipple's disease, WD）是一种易复发、少见的多系统感染性疾病，临床表现非特异，包括胃肠炎、关节炎、脑炎、心内膜炎等，但仅以呼吸道症状为主的病例较为罕见^[6]^。TW肺炎由于发病率低、表现非特异、培养率低，常常被漏诊、误诊，导致治疗的延误。近年来聚合酶链反应（polymerase chain reaction, PCR）及mNGS技术的发展使其诊断敏感性提高，有助于指导严重肺部感染患者的针对性治疗^[7]^。

### 2.2 鉴别诊断

肺部肿瘤的鉴别诊断极为重要，目前主要通过病理检查、基因检测、免疫组织化学染色等方法，结合临床表现和影像学检查来进行确诊。PPMA的临床表现无明显特异性，可出现咳嗽、气短、胸闷、呼吸困难等症状，CT表现为弥漫性肺炎型或不规则团块影，也可伴空泡征、支气管充气征、血管造影征，主要与肺炎、肺结核、消化道转移癌、其他非黏液性腺癌等相鉴别。BSCP患者因肿瘤大小、生长部位而具有不同临床表现，有复发或恶性转化的可能性，因此在诊断时应广泛取材、仔细观察，在临床上需要持续监测疾病是否进展或恶性转化^[8]^。对于肺内多发结节的诊断需谨慎，文献^[9]^指出按照标准流程基于详细的临床、分子、病理数据的全面评估是鉴别诊断的有效方法，积极进行辅助检查有助于避免误诊和漏诊。本例患者两种肿瘤分化良好、诊断明确，可以证明PPMA和BSCP是发生在同侧肺组织中独立的双原发性肿瘤，而不是肿瘤的肺内转移，并且我们考虑BSCP对支气管的堵塞可能加重了肺部感染以及PPMA的肺内转移和黏液堆积，导致症状和影像学表现更加严重。

### 2.3 治疗与分子病理特征

多原发肺肿瘤与肺内转移瘤的治疗策略和预后有差异，由于原发肿瘤组织学类型不同，因此需要根据病理、免疫、基因等多方面考虑给患者制定个体化、合理化的治疗。PPMA的治疗方法主要有局部切除术、化疗或放疗，但尚无完全有效的治疗方案，对单发局限型病变以手术为主，而弥漫性病变以化疗为主。免疫治疗和抗血管生成治疗已被认为是治疗非小细胞肺癌的广受好评的选择^[10]^，如表皮生长因子受体酪氨酸激酶抑制剂（epidermal growth factor receptor-tyrosine kinase inhibitors, EGFR-TKIs）和间变性淋巴瘤激酶（anaplastic lymphoma kinase, ALK）抑制剂。然而，文献报道鼠类肉瘤病毒癌基因（kirsten rat sarcoma, KRAS）突变与PPMA有密切的关系^[11]^，而PPMA患者EGFR突变发生率低，也有病例存在如神经调节蛋白1（neuregulin 1, NRG1）、B-RAF原癌基因丝氨酸/苏氨酸蛋白激酶（B-Raf proto-oncogene, serine/threonine kinase, BRAF）、erb-b2受体酪氨酸激酶2（erb-b2 receptor tyrosine kinase 2, ERBB2）的突变^[12]^，能否作为可靶向位点尚需进一步探究。有研究^[13]^表明BSCP恶性转化的风险与吸烟状况、年龄>40岁以及感染HR-HPV相关，完全手术切除是孤立性BSCP最常用的治疗方法，为减缓复发可进行病灶内注射西多福韦、干扰素或口服吲哚-3-甲醇的辅助药物治疗。本例患者长期大量吸烟是肺腺癌的危险因素，咳嗽、咳痰病史较长，然并未重视，若能进行常规体检可能有助于疾病的早期诊治和预后的改善。对于多原发肺部肿瘤合并感染的患者，我们考虑应当明确病因后结合患者整体情况，以控制疾病进展和改善生活质量为目标，积极抗感染治疗改善患者一般情况，然后对肺部肿瘤分主次进行针对性治疗，辅以营养支持等一般治疗，从而有效提高患者预后。

TW感染更易发生在免疫力低下的人群中^[14]^，本例患者有肺肿瘤及糖尿病病史，其症状与影像表现非特异，其他实验室检查均为阴性，最终通过肺泡灌洗液mNGS检测出TW。TW肺炎治疗尚未有明确共识，治疗经验中常使用多种药物联合，包括美罗培南+多西环素、美罗培南+磺胺甲噁唑片等，头孢曲松、哌拉西林他唑巴坦、青霉素、链霉素也对治疗有效^[15,16]^。甲氧苄啶/磺胺甲噁唑能透过血脑屏障且有效降低复发，也有报道^[17,18]^称其使用与耐药相关而推荐使用磺胺嘧啶。本病例初期经验性使用厄他培南后症状未明显改善，入院8天后加用哌拉西林钠他唑巴坦钠，入院15天后复查CT感染范围仍较大，继续使用7天后患者咳嗽咳痰症状减轻，一般情况尚可，予以出院，我们考虑该患者抗感染效果不佳可能与合并肺肿瘤、免疫力低下、耐药等有关，并且患者因过敏不能使用推荐用药头孢曲松可能也影响了治疗效果。TW感染引起的WD会累及多系统，该患者并未出现关节、肠道、心血管、皮肤的症状，其神志淡漠、记忆力减退的症状是否与TW感染有关尚不明确，在随访过程中患者多次因健忘、失眠、淡漠等神经症状就诊，头颅核磁检查示脑白质病变（Fazekas 3级）与筛窦炎症，然而遗憾的是由于患者基本情况较差我们未能进行脑脊液或PCR检测。因此，对于重症肺炎应积极寻找病原学证据并根据患者具体情况进行调整用药，肺部TW的检出还需要结合临床进行，分析病原与疾病的关联性，在没有条件进一步验证时我们仍建议在用药时覆盖TW以尽早控制感染。

综上所述，本文报道了1例PPMA合并BSCP及TW感染的罕见病例，让我们对多发肺肿瘤以及TW肺炎的诊疗有了更加深刻的认识，在鉴别诊断时应综合考虑各项检查结果与病理指标，排除合并多种病变的可能性，后续治疗也应制定符合患者的个性化方案。

**Competing interests**

The authors declare that they have no competing interests.

## References

[1] Sung H, Ferlay J, Siegel RL, et al. Global cancer statistics 2020: GLOBOCAN estimates of incidence and mortality worldwide for 36 cancers in 185 countries. CA Cancer J Clin, 2021, 71(3): 209-249. doi: 10.3322/caac.21660 33538338

[2] Nie K, Nie W, Zhang YX, et al. Comparing clinicopathological features and prognosis of primary pulmonary invasive mucinous adenocarcinoma based on computed tomography findings. Cancer Imaging, 2019, 19(1): 47. doi: 10.1186/s40644-019-0236-2 31292000 PMC6617846

[3] Popper HH, Wirnsberger G, Jüttner-Smolle FM, et al. The predictive value of human papilloma virus (HPV) typing in the prognosis of bronchial squamous cell papillomas. Histopathology, 1992, 21(4): 323-330. doi: 10.1111/j.1365-2559.1992.tb00402.x 1328017

[4] Travis WD, Brambilla E, Noguchi M, et al. International Association for the Study of Lung Cancer/American Thoracic Society/European Respiratory Society international multidisciplinary classification of lung adenocarcinoma. J Thorac Oncol, 2011, 6(2): 244-285. doi: 10.1097/JTO.0b013e318206a221 21252716 PMC4513953

[5] Goodwin D, Rathi V, Conron M, et al. Genomic and clinical significance of multiple primary lung cancers as determined by next-generation sequencing. J Thorac Oncol, 2021, 16(7): 1166-1175. doi: 10.1016/j.jtho.2021.03.018 33845213

[6] Lin M, Wang K, Qiu L, et al. Tropheryma whipplei detection by metagenomic next-generation sequencing in bronchoalveolar lavage fluid: A cross-sectional study. Front Cell Infect Microbiol, 2022, 12: 961297. doi: 10.3389/fcimb.2022.961297 36061864 PMC9428251

[7] Zhang WM, Xu L. Pulmonary parenchymal involvement caused by Tropheryma whipplei. Open Med (Wars), 2021, 16(1): 843-846. doi: 10.1515/med-2021-0297 34131590 PMC8174119

[8] Manley C, Hutchinson C, Mahajan A, et al. Treatment of recurrent respiratory papillomatosis: case series and review of technique. Surg Technol Int, 2021, 38: 139-143. doi: 10.52198/21.STI.38.GS1408 33844241

[9] Girard N, Deshpande C, Lau C, et al. Comprehensive histologic assessment helps to differentiate multiple lung primary non-small cell carcinomas from metastases. Am J Surg Pathol, 2009, 33(12): 1752-1764. doi: 10.1097/PAS.0b013e3181b8cf03 19773638 PMC5661977

[10] Zhou D, Gulinuer W, Zhu N. Chemotherapy in combination with pembrolizumab and antiangiogenesis in young patients with advanced primary pulmonary mucinous adenocarcinoma: Two case reports. Sci Prog, 2021, 104(4): 368504211061971. doi: 10.1177/00368504211061971 PMC10461373 34842490

[11] Izumi M, Oyanagi J, Sawa K, et al. Mutational landscape of multiple primary lung cancers and its correlation with non-intrinsic risk factors. Sci Rep, 2021, 11(1): 5680. doi: 10.1038/s41598-021-83609-y 33707471 PMC7952588

[12] Shim HS, Kenudson M, Zheng Z, et al. Unique genetic and survival characteristics of invasive mucinous adenocarcinoma of the lung. J Thorac Oncol, 2015, 10(8): 1156-1162. doi: 10.1097/JTO.0000000000000579 26200269

[13] Himuro N, Niiya Y, Minakata T, et al. A solitary bronchial squamous cell papilloma with increased 18-fluorodeoxyglucose uptake and high serum levels of squamous cell carcinoma antigen. J Thorac Dis, 2018, 10(6): E435-E437. doi: 10.21037/jtd.2018.05.78 30069399 PMC6051801

[14] Marth T, Raoult D. Whipple’s disease. Lancet, 2003, 361(9353): 239-246. doi: 10.1016/S0140-6736(03)12274-X 12547551

[15] Li W, Zhang Q, Xu Y, et al. Severe pneumonia in adults caused by Tropheryma whipplei and Candida sp. infection: a 2019 case series. BMC Pulm Med, 2021, 21(1): 29. doi: 10.1186/s12890-020-01384-4 33451316 PMC7810182

[16] Yin XD, Xu ML. Severe Tropheryma whipplei pneumonia: a case report. Zhongguo Ganran Kongzhi Zazhi, 2022, 21(8): 812-815.

[17] Zhang HM, Yu HY, Zou M, et al. Analysis of clinical features of Tropheryma whipplei pneumonia. Huaxi Yixue, 2023, 38(4): 500-505.

[18] Puéchal X. Whipple's disease. Ann Rheum Dis, 2013, 72(6): 797-803. doi: 10.1136/annrheumdis-2012-202684 23291386

